# Stratified neutrophil-to-lymphocyte ratio accurately predict mortality risk in hepatocellular carcinoma patients following curative liver resection

**DOI:** 10.18632/oncotarget.6707

**Published:** 2015-12-21

**Authors:** Gui-Qian Huang, Gui-Qi Zhu, Yan-Long Liu, Li-Ren Wang, Martin Braddock, Ming-Hua Zheng, Meng-Tao Zhou

**Affiliations:** ^1^ Department of Infection and Liver Diseases, Liver Research Center, the First Affiliated Hospital of Wenzhou Medical University, Wenzhou 325000, China; ^2^ Renji School of Wenzhou Medical University, Wenzhou 325000, China; ^3^ School of the First Clinical Medical Sciences, Wenzhou Medical University, Wenzhou 325000, China; ^4^ College of Pharmaceutical Sciences, Wenzhou Medical University, Wenzhou, 325035, China; ^5^ Global Medicines Development, AstraZeneca R&D, Alderley Park, United Kingdom; ^6^ Institute of Hepatology, Wenzhou Medical University, Wenzhou 325000, China; ^7^ Department of Hepatobiliary Surgery, the First Affiliated Hospital of Wenzhou Medical University, Wenzhou 325000, China

**Keywords:** neutrophil-to-lymphocyte ratio, hepatocellular carcinoma, curative liver resection, overall survival

## Abstract

**Objectives:**

Neutrophil lymphocyte ratio (NLR) has been shown to predict prognosis of cancers in several studies. This study was designed to evaluate the impact of stratified NLR in patients who have received curative liver resection (CLR) for hepatocellular carcinoma (HCC).

**Methods:**

A total of 1659 patients who underwent CLR for suspected HCC between 2007 and 2014 were reviewed. The preoperative NLR was categorized into quartiles based on the quantity of the study population and the distribution of NLR. Hazard ratios (HRs) and 95% confidence intervals (CIs) were significantly associated with overall survival (OS) and derived by Cox proportional hazard regression analyses. Univariate and multivariate Cox proportional hazard regression analyses were evaluated for association of all independent parameters with disease prognosis.

**Results:**

Multivariable Cox proportional hazards models showed that the level of NLR (HR = 1.031, 95%CI: 1.002-1.060, P = 0.033), number of nodules (HR = 1.679, 95%CI: 1.285-2.194, P<0.001), portal vein thrombosis (HR = 4.329, 95%CI: 1.968-9.521, P<0.001), microvascular invasion (HR = 2.527, 95%CI: 1.726-3.700, P<0.001) and CTP score (HR = 1.675, 95%CI: 1.153-2.433, P = 0.007) were significant predictors of mortality. From the Kaplan-Meier analysis of overall survival (OS), each NLR quartile showed a progressively worse OS and apparent separation (log-rank P=0.008). The highest 5-year OS rate following CLR (60%) in HCC patients was observed in quartile 1. In contrast, the lowest 5-year OS rate (27%) was obtained in quartile 4.

**Conclusions:**

Stratified NLR may predict significantly improved outcomes and strengthen the predictive power for patient responses to therapeutic intervention.

## INTRODUCTION

Hepatocellular carcinoma (HCC) is the third most common cause of mortality in the world and at least 300,000 of the 600,000 deaths worldwide occur in China alone [1]. Some population-based studies show that the incidence rate of HCC continues to approximate to the death rate, suggesting that the majority of patients with HCC die with from this disease [2–3]. At present, curative liver resection (CLR) provides a radical therapy in patients with early stages of the disease, but is associated with a high-risk of recurrence and a poor long-term prognosis [4–6]. Therefore, it is necessary monitor patients for progression of HCC to reduce the recurrence rate and to prolong the survival period in HCC patients after CLR.

Currently, several studies indicate that genetic, environmental and biological factors are contributory risk factors for the development and progression of HCC [1–2, 7]. In addition, a number of clinicopathologic features have been identified as prognostic indicators for HCC patients, such as vascular invasion, tumor size, the level of serum a-fetoprotein (AFP) and bilirubin [8–11]. Of particular interest, recent studies show that systemic inflammatory responses lead to the promotion of angiogenesis, DNA damage, and tumor invasion through the upregulation of cytokines in many cancers [12–15]. The neutrophil—lymphocyte ratio (NLR), a marker of systemic inflammation, is a simple ratio of the absolute neutrophil and lymphocyte counts from the differential component of the blood leukocyte count, and it appears to perform a better prognosis of disease in patients with breast, gastric, lung, and rectal cancers [16–19]. Furthermore, an elevated level of pre-procedural NLR has shown a significant correlation with a poorer prognosis in patients undergoing liver transplantation for HCC [20], and a preoperative NLR ≥ 5 is an adverse predictor of disease-free and overall survival in HCC patients after curative resection [21]. Among patients with Hepato-pancreatico-biliary malignancy undergoing resection, elevated NLR is also a predictor of worse long-term outcome [22].

These studies have shown that the preoperative NLR has been a useful and informative prognostic marker in advanced diseases, including HCC. However, to date, there have been no reports regarding NLR in HCC patients undergoing CLR with stratification to predict overall survival. The main aim of this study was to construct the stratification with NLR to enhance the prognostic utility for patients who underwent CLR for suspected HCC.

## RESULTS

### Baseline characteristics of all patients in NLR quartiles

**Figure 1:**
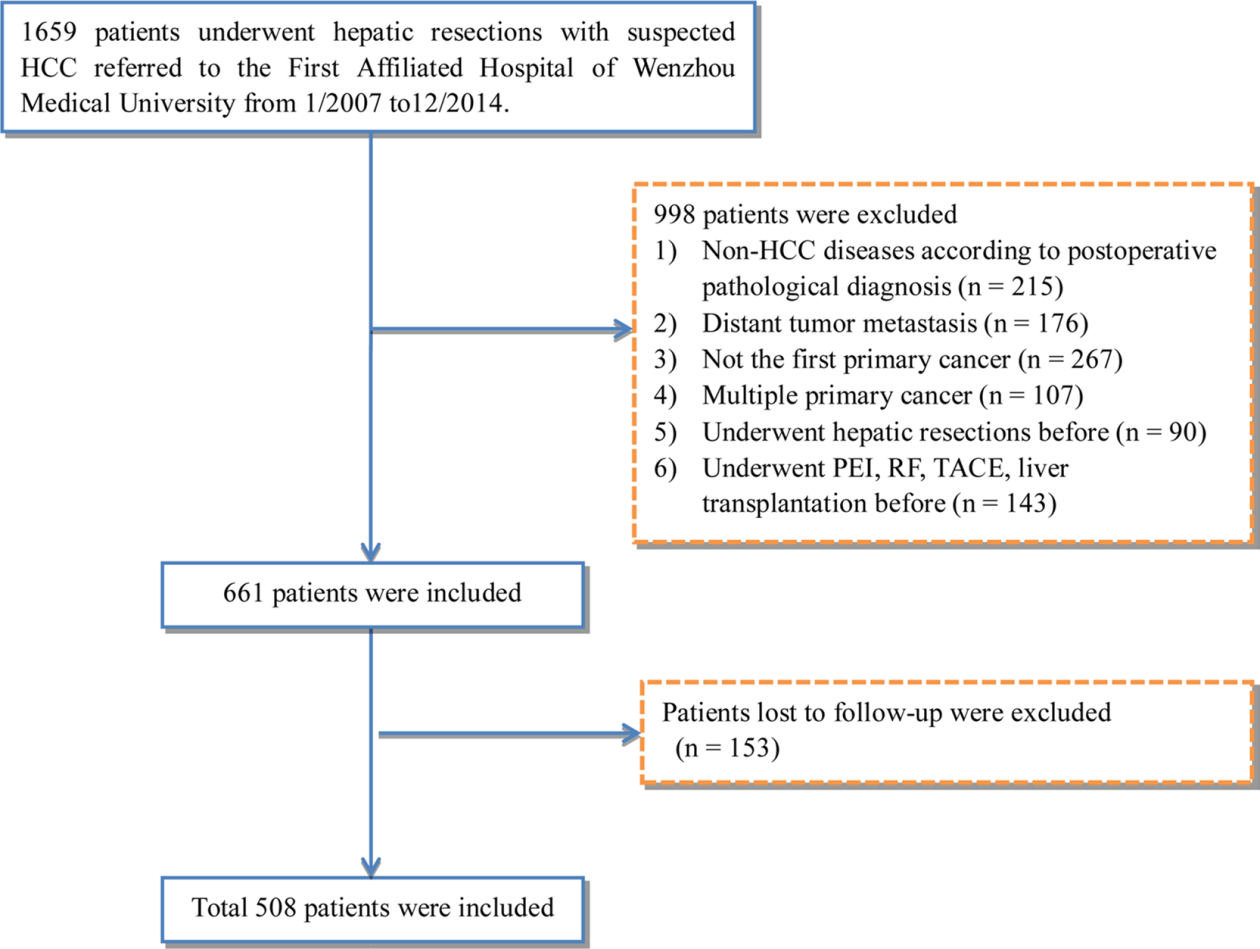
Study flow diagram HCC, hepatocellular carcinoma; PEI, percutaneous ethanol injection; RF, radiofrequency; TACE, transarterial chemoembolization.

508 patients meeting the inclusion criteria from 1659 patients who received CLR for suspected HCC were selected into this study and were consisted of 432 males and 76 females with a mean age of 56.5 ± 10.9 years (range, 23 to 85) (Figure 1, Table 1). The majority of patients were male (85%), and Hepatitis B virus was the main etiology in this study (67.9%). According to the quartiles of NLR, all of the patients were divided into four groups, because this method ensured the most categories with adequate number of patients per category from the range of 0.54 to 38.5 (127 patients per group). The cut-off points of this stratification were: (Q1) 0.54-1.67, (Q2) 1.67-2.33, (Q3) 2.33-3.83 and (Q4) 3.83-38.5. The correlation of demographic, clinical, tumor and laboratory characteristics with NLR quartiles were shown in Table 1. There was no difference in the incidence of major complications and the demographic parameters among patients (all P > 0.05). Furthermore, patients with low and high NLR seemed to similar with regard to performance of laboratory characteristics except for total bilirubin, direct bilirubin, albumin, aspartate aminotransferase (AST), blood glucose, prothrombin time (PT), prothrombin time activity (PTA), INR and white blood cell. Because there were fever numbers of patients in each quartile, some significant difference of baseline characteristics is reasonable.

**Table 1:** Characteristics of patients with hepatocellular carcinoma treated by surgical resection according to NLR quartiles

Variables	All patients	NLR quartiles				
		Quartile 1 n = 127 (0.54-1.67)	Quartile 2 n = 127 (1.67-2.33)	Quartile 3 n = 127 (2.33-3.83)	Quartile 4 n = 127 (3.83-38.5)	P-value
NLR	2.3 (1.7, 3.8)	1.3 (1.1, 1.5)	2 (1.8, 2.2)	2.9 (2.5, 3.3)	7.5 (5.2, 12.4)	<0.001
**Demographic parameters**						
Age (years)	56.5 ± 10.9	55.4 ± 10.6	56.6 ± 10.3	56.7 ± 10.9	57.2 ± 12.0	0.594
Gender						0.613
Male	432 (85.0%)	105 (82.7%)	108 (85.0%)	106 (83.5%)	113 (89.0%)	
Female	76 (15.0%)	22 (17.3%)	19 (15.0%)	21 (16.5%)	14 (11.0%)	
BMI (kg/m^2^)	22.8 (20.8, 24.6)	22.9 (21.1, 24.5)	23.1 (21.1, 24.6)	22.8 (20.9, 24.8)	22 (20.3, 24.5)	0.550
**Clinical parameters**						
Ascites, n (%)						0.091
Absence	386 (90.2%)	105 (92.1%)	102 (92.7%)	105 (93.8%)	74 (80.4%)	
Presence	42 (9.8%)	9 (7.9%)	8 (7.3%)	7 (6.2%)	18 (19.6%)	
Liver cirrhosis, n (%)	186 (42.8%)	48 (41.7%)	43 (38.4%)	54 (47.8%)	41 (43.2%)	0.480
**Etiology**						<0.001
Hepatitis B, n (%)	341 (67.9%)	96 (76.1%)	90 (70.9%)	81 (63.8%)	74 (60.7%)	
Alcohol, n (%)	40 (8.0%)	7 (5.6%)	8 (6.3%)	13 (10.2%)	12 (9.8%)	
Hepatitis B + Hepatitis C, n (%)	80 (15.9%)	19 (15.1%)	21 (16.5%)	20 (15.7%)	20 (16.4%)	
Other, n (%)	38 (7.6%)	4 (3.2%)	7 (5.5%)	12 (9.4%)	15 (12.3%)	
Hepatitis C, n (%)	3 (0.6%)	0	1 (0.8%)	1 (0.9%)	1 (0.8%)	
**Laboratory parameters**						
Total bilirubin (μmol/L)	10.0 (8.0, 15.0)	10.0 (8.0, 14.0)	9.0 (7.0, 14.0)	10.0 (7.0, 14.0)	13.0 (10.0, 22.0)	<0.001
Direct bilirubin (μmol/L)	4.0 (2.8, 6.0)	3.0 (2.0, 5.0)	3.0 (2.0, 5.0)	3.0 (2.8, 5.0)	5.0 (3.0, 8.5)	<0.001
Albumin (g/L)	40.5 (37.3, 43.6)	39.9 (36.8, 43.2)	41.3 (38.2, 44.3)	41.6 (38.5, 44.2)	39.2 (35.1, 42.3)	<0.001
ALT (IU/L)	36.0 (25.0, 55.0)	38.0 (28.0, 50.0)	35.0 (25.0, 54.0)	34.0 (23.0, 53.0)	37.0 (24.8, 65.3)	0.422
AST (IU/L)	37.0 (27.0, 54.0)	36.0 (29.0, 53.0)	34.0 (26.5, 49.5)	33.0 (24.0, 51.0)	41.0 (30.0, 85.0)	0.004
Alkaline phosphatase (IU/L)	95.0 (75.0, 116.0)	89.5 (74.3, 108.8)	98.0 (76.3, 114.0)	93.5 (73.3, 122.8)	98.5 (76.0, 135.0)	0.326
γ-GT (IU/L)	59.5 (34.3, 110.5)	54.0 (34.3, 92.8)	58.0 (30.3, 119.3)	52.5 (33.0, 126.0)	71.5 (38.5, 124.8)	0.242
Blood glucose (mmol/L)	5.9 (5.0, 7.3)	5.4 (4.8, 6.6)	6.0 (5.1, 7.8)	6.2 (5.2, 7.9)	6.0 (5.1, 7.9)	<0.001
Creatinine (μmol/L)	67.0 (56.0, 76.0)	67.0 (57.0, 77.0)	67.0 (57.0, 76.0)	66.0 (54.0, 74.0)	66.5 (57.0, 79.0)	0.626
Serum sodium (mmol/L)	141.0 (139.0, 142.0)	141.0 (139.0, 143.0)	141.0 (139.0, 143.0)	140.0 (139.0, 142.0)	140.5 (138.0, 142.3.0)	0.671
PT (s)	13.9 (13.3, 14.8)	14.0 (13.3, 14.9)	13.7 (13.2, 14.4)	13.8 (13.2, 14.4)	14.2 (13.6, 15.2)	<0.001
PTA (%)	87.9 ± 13.8	85.6 ± 14	91.1 ± 12.8	91 ± 11.4	83.9 ± 15.3	<0.001
INR	1.1 (1.0, 1.2)	1.1 (1.0, 1.2)	1.1 (1.0, 1.1)	1.1 (1.0, 1.1)	1.1 (1.0, 1.2)	<0.001
White blood cell (10^9^/L)	5.3 (4.2, 6.7)	4.7 (3.8, 5.5)	5.4 (4.3, 6.3)	5.5 (4.5, 6.9)	5.9 (4.4, 8.8)	<0.001
AFP (ng/mL)	30.4 (5.5, 452.2)	29.6 (5.4, 285.5)	51.1 (6.1, 943)	22.9 (5.0, 469.1)	28.6 (4.9, 413.6)	0.506
Uric acid (μmol/L)	300.4 ± 91.4	312.4 ± 87.5	300.5 ± 86.1	294.7 ± 81	293.9 ± 108.5	0.342
Platelet (10^9^/L)	140.3 ± 66.0	131.0 ± 56.1	146.1 ± 62.4	147.5 ± 67.0	136.9 ± 76.1	0.128
**Tumor Characteristics**						
Number of nodules						0.007
1	424 (87.2%)	108 (87.8%)	109 (87.2%)	106 (84.8%)	101 (89.4%)	
2	41 (8.4%)	10 (8.1%)	11 (8.8%)	13 (10.4%)	7 (6.2%)	
3	9 (1.9%)	2 (1.6%)	2 (1.6%)	2 (1.6%)	3 (2.7%)	
≥4	12 (2.5%)	3 (2.4%)	3(2.4%)	4 (3.2%)	2 (1.8%)	
Greatest tumor diameter (mm)	49.1 ± 33.0	37.1 ± 22.5	49.6 ± 29.6	52.1 ± 38.5	58.4 ± 36.1	<0.001
Portal vein thrombosis, n (%)	15 (3.5%)	1 (0.9%)	4 (3.6%)	5 (4.5%)	5 (5.4%)	0.042
Microvascular invasion, n (%)	133 (26.4%)	38 (30.2%)	27 (21.4%)	28 (22.2%)	40 (31.7%)	<0.001
**CLIP score**						<0.001
0, n (%)	182 (44.5%)	62 (55.9%)	40 (38.8%)	53 (48.1%)	27 (31.7%)	
1, n (%)	87 (21.3%)	25 (22.5%)	25 (24.3%)	20 (18.2%)	17 (20.0%)	
2, n (%)	73 (17.8%)	16 (14.4%)	19 (18.4%)	20 (18.2%)	18 (21.2%)	
3, n (%)	51 (12.5%)	4 (3.6%)	15 (14.6%)	17 (15.5%)	15 (17.6%)	
4, n (%)	14 (3.4%)	4 (3.6%)	4 (3.9%)	0	6 (7.1%)	
5, n (%)	2 (0.5%)	0	0	0	2 (2.4%)	
**CTP score**						0.031
A, n (%)	352 (83.0%)	97 (85.1%)	93 (85.3%)	101 (91.0%)	61 (67.8%)	
B, n (%)	64 (15.1%)	14 (12.3%)	15 (13.8%)	9 (8.1%)	26 (28.9%)	
C, n (%)	8 (1.9%)	3 (2.6%)	1 (0.9%)	1 (0.9%)	3 (3.3%)	
**Follow-up data**						
Death within 36 months of resection						<0.001
Alive	145 (54.9%)	34 (56.7%)	34 (61.8%)	40 (60.6%)	37 (44.6%)	
Deceased	119 (45.1%)	26 (43.3%)	21 (38.2%)	26 (39.4%)	46 (55.4%)	

**NOTE**. NLR, neutrophil-lymphocyte ratio; ALT, alanine aminotransferase; AST, aspartate aminotransferase; γ-GT, γ-glutamyl transferase; PT, prothrombin time; PTA, prothrombin time activity; AFP, alpha-fetoprotein; INR, international normalized ratio; CLIP, Cancer of The Liver Italian Program; CTP, Child-Turcotte-Pugh.

### Survival analysis

**Figure 2:**
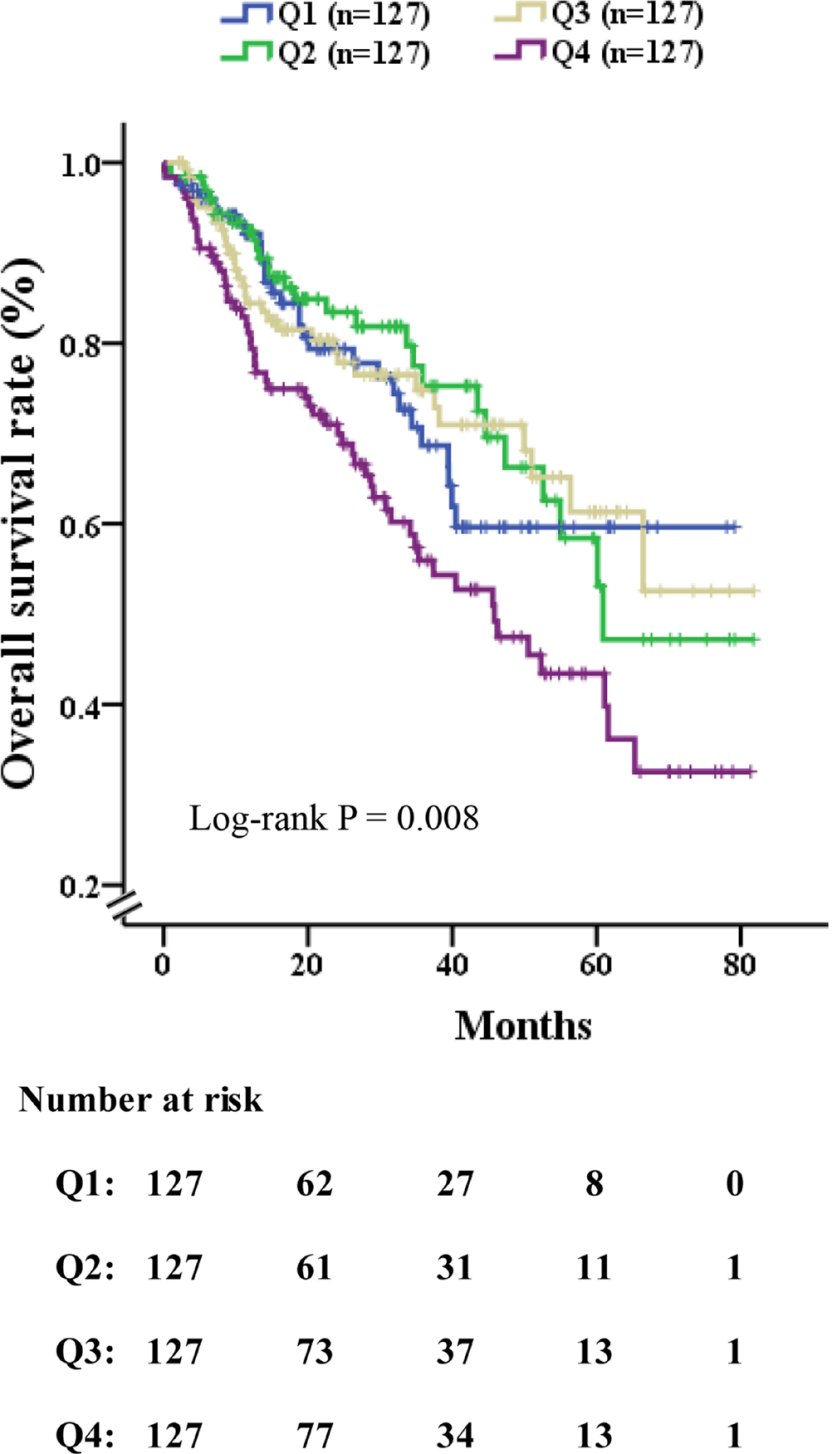
Overall survival rate of patients who had received curative liver resection, stratified by quartile of NLR The log-rank P value among all four quartiles was 0.008. (Q1) 0.54-1.67, (Q2) 1.67-2.33, (Q3) 2.33-3.83 and (Q4) 3.83-38.5. Patients with highest NLR (Q1) had favorable 5-year survival following surgery (60%), however, those in the lowest quartile of NLR (Q4) had poor outcomes (27%).

Table 2 showed the univariate and multivariate Cox proportional hazards analyses in all patients. On the univariate Cox proportional hazards analysis, factors associated with mortality included the NLR, albumin, alanine aminotransferase, blood glucose, ascites, AST, alkaline phosphatase (AKP), γ-glutamyl transferase (γ-GT), PT, PTA, AFP, white blood cell, platelet, number of nodules, greatest tumor diameter, MVI, portal vein thrombosis, CTP score and CLIP score (all P < 0.05). After extensive univariate analysis, these significant variables were included in the multivariable Cox proportional hazards models, which showed that the level of NLR (HR = 1.031, 95%CI: 1.002-1.060, P = 0.033), number of nodules (HR = 1.679, 95%CI: 1.285-2.194, P<0.001), portal vein thrombosis (HR = 4.329, 95%CI: 1.968-9.521, P<0.001), microvascular invasion (HR = 2.527, 95%CI: 1.726-3.700, P<0.001) and CTP score (HR = 1.675, 95%CI: 1.153-2.433, P = 0.007) were significant predictors of mortality. From the Kaplan-Meier analysis of OS with patients (Figure 2), each quartile performed the difference of OS apparently (log-rank P=0.008), patients with the lowest quartile of NLR (Q1) had very favorable 5-year OS following CLR (60%), however, those in the highest quartile of NLR (Q4) poor outcomes (27%)

**Table 2:** Univariate and multivariate Cox Proportional Hazards Regression Analyses of factors associated with Mortality

Variables	Univariate analysis				Multivariate analysis			
	B	HR	95%CI	P-value	B	HR	95%CI	P-value
NLR	0.039	1.040	1.015-1.065	0.001	0.031	1.031	1.002-1.060	0.033
**Demographic parameters**								
Age (years)	0.008	1.008	0.993-1.023	0.284				
Gender	−0.036	0.965	0.602-1.547	0.881				
BMI	0.005	1.005	0.952-1.06	0.865				
**Clinical parameters**								
Ascites	0.000	1.000	1.000-1.000	0.007				
Liver cirrhosis	0.198	1.219	0.852-1.746	0.279				
**Laboratory parameters**								
Total bilirubin (μmol/L)	0.005	1.005	1.000-1.010	0.057				
Direct bilirubin (μmol/L)	0.006	1.006	0.999-1.012	0.101				
Albumin (g/L)	−0.061	0.941	0.917-0.966	<0.001				
ALT (IU/L)	0.002	1.002	1.000-1.003	0.041				
AST (IU/L)	0.001	1.001	1.000-1.002	0.005				
Alkaline phosphatase (IU/L)	0.002	1.002	1.001-1.003	0.006				
γ-GT (IU/L)	0.001	1.001	1.000-1.002	0.047				
Blood glucose (mmol/L)	0.046	1.047	1.004-1.092	0.032				
Creatinine (μmol/L)	−0.004	0.996	0.987-1.005	0.408				
Uric acid (μmol/L)	0.000	1.000	0.998-1.002	0.978				
Serum sodium (mmol/L)	0.003	1.003	0.998-1.008	0.276				
PT (s)	0.152	1.164	1.039-1.305	0.009				
PTA (%)	−0.015	0.985	0.973-0.997	0.013				
INR	0.008	1.008	0.988-1.028	0.433				
White blood cell (10^9^/L)	0.025	1.025	1.009-1.041	0.002				
Platelet (10^9^/L)	0.003	1.003	1.000-1.005	0.038				
AFP (ng/mL)	0.000	1.000	1.000-1.000	0.007				
**Tumor Characteristics**								
Number of nodules	0.423	1.526	1.225-1.901	<0.001	0.518	1.679	1.285-2.194	<0.001
Greatest tumor diameter (mm)	0.010	1.010	1.005-1.014	<0.001				
Portal vein thrombosis	1.778	5.919	2.835-12.358	<0.001	1.465	4.329	1.968-9.521	<0.001
Microvascular invasion	0.897	2.453	1.770-3.399	<0.001	0.927	2.527	1.726-3.700	<0.001
**CLIP score**	0.445	1.560	1.342-1.813	<0.001				
**CTP score**	0.691	1.996	1.418-2.810	<0.001	0.516	1.675	1.153-2.433	0.007

NOTE. B, coefficient; HR, hazard ratio; CI, confidence interval; NLR, neutrophil-lymphocyte ratio; ALT, alanine aminotransferase; AST, aspartate aminotransferase; γ-GT, γ-glutamyl transferase; PT, prothrombin time; PTA, prothrombin time activity; AFP, alpha-fetoprotein; INR, international normalized ratio; CLIP, Cancer of The Liver Italian Program; CTP, Child-Turcotte-Pugh.

## DISCUSSION

In this study, the stratification of NLR level was first established to predict 36-month prognosis of patients who underwent CLR for HCC. Based on Kaplan-Meier analysis of OS, the elevated level of NLR was associated with the poor survival of liver cancer and high quartile of NLR was associated with poor prognosis.

The association between tumors and inflammation was discovered over a century ago [23]. However, the mechanism by which the immune response may be triggered by a tumor is complex [24] and this has stimulated research for an underlying mechanism that associates tumor inflammation and disease prognosis [13,25]. NLR was frequently used as an inflammatory marker, and its’ prognostic role in liver cancer has recently been described [20,26–28]. The recent meta-analyses have confirmed the prognostic value of the NLR in HCC [29]. In addition, surgery in cancer patients has been shown to influence lymphocyte function, including reducing lymphocyte adenosine triphosphate production following hysterectomy, colostomy, and also after blood transfusion [30]. This was also been observed in HCC patients after CLR, some studies indicate that pre-operative NLR was an adverse predictor of disease-free and overall survival in these patients [21,31]. As the level of NLR is the widely accepted serum biomarker to diagnose cancer and predict the recurrence of cancer, we first stratified NLR to predict prognosis in patients who underwent CLR for HCC and to ask whether this could be used to predict a better performance. We found that the presence of elevated pre-operative NLR was associated with poor survival, which is consistent with the study of Walsh et al. who reported that NLR has been shown to be a bio- marker of inflammation and of prognostic significance in colorectal carcinoma [24]. Furthermore, Zhao et al. demonstrated that an elevated level of NLR was associated with poor survival in patients with lung cancer [32].

The relationship between elevated pre-operative NLR and a poor prognosis is complex and remains to be elucidated. One possible explanation is that as part of a paraneoplastic syndrome, the tumor will produce myeloid growth factors leading to an increased production of neutrophils. For instance, granulocyte colony-stimulating factor, causes neutrophilia by acting specifically on bone marrow granulocytic cells [23,33–35]. An alternative explanation may be that a raised level of neutrophils may aid in the development of the neoplasm through providing an adequate environment for growth and proliferation [21]. In support of this, Kusumanto et al. found that the total circulating level of vascular endothelial growth factor (VEGF), a pro-angiogenic growth factor, is contained in granulocytes, especially in the neutrophils, which is thought to be involved in tumor development [36].

Recently, a number of studies in oncology have explored whether a better effect on disease prognosis can be achieved by stratification of an independent predictor, such as categorizing AFP into quintiles, creating the opportunity to observe differences in outcomes among HBV-HCC patients following surgical resection [37]. In this study, in view of the fact that the level of NLR is a widely accepted HCC risk factor, we categorized NLR into quartiles to investigate whether any enhanced predictive affect was detected. Consequently, we gained greater confidence in being able to predict clinical outcome, which was illustrated by a favorable outcome (the lowest quartile of NLR, with a 5-year survival of 60%) and a poor outcome (the highest quartile of NLR, with a 5-year survival of 27%). These new categories have shown distinct and significant survival outcomes in HCC patients, and so this may be helpful in guiding the clinician to predict the prognosis of disease and then to select the most appropriate treatment or palliative care. Hence, our study suggests that the stratification of NLR could independently contribute to the disease prognosis following CLR for suspected HCC.

The current study has several limitations. Our study requires further studies to further validate the performance of the stratification of NLR. Moreover, the findings may not be applicable to HCC patients who receive other therapies or surgeries [38], and data from further large-scale clinical researches are needed to evaluate the effect of categorizing NLR on patients who underwent CLR for suspected HCC. Finally, more predictive markers, such as the newly peritumoral Cbl and Na^+^/K^+^-ATPase α_1_ subunit [39–40], should be integrated into the prognostic system in the future.

In conclusion, we show for the first time a categorization of patients with pre-operative NLR into quartiles and that we may be able to predict significantly improved outcomes among HCC patients following CLR. We suggest that clinicians should consider the level of NLR to help select the most appropriate therapy plan for their patients with HCC.

## MATERIALS AND METHODS

### Study design

In this study, all patients were sampled consecutively from CLR records for suspected HCC between January 2007 and January 2014 at the First Affiliated Hospital of Wenzhou Medical University. All cases of suspected HCC were confirmed by pathological analysis.

NLR was calculated by dividing the absolute level of neutrophils by the absolute level of lymphocytes on the basis of preoperative blood values. Furthermore, based on the quantity of the study population and the distribution of NLR with greatest differences in patient outcomes following surgery, NLR was further categorized into quartiles to investigate whether any enhanced predictive affect was detected while maintaining sufficient statistical power in each category. The study was performed according to Standards for the Reporting of Diagnostic Accuracy Studies, and it was approved by the Committee on the Ethics at the First Affiliated Hospital of Wenzhou Medical University while the written informed consents were obtained from all patients before the initiation of the study.

### Exclusion criteria

For selection of patients into our analyses, the following exclusion criteria were used as follows: (1) non-HCC disease on the basis of post-operative pathological diagnosis; (2) distant tumor metastasis; (3) not the first primary cancer; (4) multiple primary tumor; (5) previous history of hepatic resection; (6) previous history of radio-frequency treatment, or trans-catheter arterial chemoembolization, liver transplantation or percutaneous ethanol injection; (7) lost to follow-up. In total, 508 cases of HCC were identified and confirmed by post-operative pathology results.

### Data collection and follow-up

Various items were abstracted from the patients’ medical records, including patient demographics, etiology of HCC, laboratory and clinical tests within the week prior to CLR. Clinical and demographic information included age, gender, BMI, laboratory tests, calculated The Cancer of the Liver Italian Program (CLIP) and Child-Turcotte-Pugh (CTP) score at initial presentation. The presence of microvascular invasion (MVI) was defined by evidence of tumor emboli in either the central hepatic vein, the portal, or the large capsular vessels on imaging studies or during surgical resection as we had previously described [41–42]. Clinical parameters such as ascites and liver cirrhosis (LC) were found by physical examination and confirmed by CT, abdominal ultrasonography or magnetic resonance imaging (MRI). Tumor characteristics included the number of tumor nodules on the basis of the CT or MRI scan, tumor size and portal vein thrombosis. Tumor size was defined as the maximal diameter of the tumor in imaging studies and portal vein thrombosis were observed during the surgery.

By March 2014 all postoperative patients were followed up once every 3 months. Death data of all-cause mortality were collected by the medical records, the social security death index as well as families, which guarantee the information of all decedents was obtained completely.

### Statistical analysis

All categorical values were presented as number and proportions and compared with the Pearson's chi-square test or Fisher's exact test as appropriate. Continuous variables were judged for distribution type by using Kolmogorov-Smirnov test and reported as mean ± standard deviation (normal distribution) or median and interquartile range (abnormal distribution). And the differences of continuous variables between groups were evaluated by the one-way analysis of variance (ANOVA) or nonparametric Kruskal-Wallis test.

Hazard ratios (HRs) and 95% confidence intervals (CIs) were significantly associated with overall survival (OS) and derived by Cox proportional hazard regression analyses. The factors identified at univariate Cox proportional hazard regression analyses were further evaluated for association of all parameters with post-operative prognostic feature, and some of these with significant differences (p < 0.05) entered into a multivariate analysis to test whether to perform the significant effects while adjusting for multiple factors simultaneously. Then, the OS rates stratified by values of NLR were calculated using the Kaplan—Meier method and compared by the log-rank test.

For all analyses, a two-tailed p value of < 0.05 was considered to be of statistical significance. All statistical analyses were performed with SPSS version 18.0 (SPSS, Chicago, IL, USA) and MedCalc version 12.7 (MedCalc Software, Ostend, Belgium).

## Abbreviation

AFP: alpha-fetoprotein
AKP: alkaline phosphatase
AST: aspartate aminotranferase
CI: confidence interval
CLR: curative liver resection
CT: computerized tomography
HCC: hepatocellular carcinoma
HR: hazard ratio
MRI: magnetic resonance imaging
LC: liver cirrhosis
MVI: microvascular invasion
NLR: neutrophil-to-lymphocyte ratio
OS: overall survival
γ-GT: γ-glutamyl transferase.
